# Supplementary material for: Regional and Urban-Scale Environmental Influences of Oceanic DMS Emissions over Coastal China Seas
Source: Atmosphere (Basel). Author manuscript; Available in PMC 2021 Aug 11. (PMC7529109; doi:10.3390/atmos11080849)
Supplement: Supplement1 [file NIHMS1619727-supplement-Supplement1.pdf]

atmosphere

MDPI

Supplementary Information

Regional and Urban-Scale Environmental Influences of Oceanic DMS Emissions over Coastal China Seas

Shanshan Li [1], Yan Zhang [1,2,3,*], Junri Zhao [1], Golam Sarwar [4], Shengqian Zhou [1], Ying Chen [1], Guipeng Yang [5] and Alfonso Saiz-Lopez [6]

[1] Shanghai Key Laboratory of Atmospheric Particle Pollution and Prevention (LAP3), Department of Environmental Science and Engineering, Fudan University, Shanghai 200438, China; shanshan_li1130@163.com (S.L.); 19110740023@fudan.edu.cn (J.Z.); 17110740002@fudan.edu.cn (S.Z.); yingchen@fudan.edu.cn (Y.C.)
[2] Big Data Institute for Carbon Emission and Environmental Pollution, Fudan University, Shanghai 200433, China
[3] Institute of Atmospheric Sciences, Fudan University, Shanghai 200438, China
[4] Center for Environmental Measurement and Modeling, Office of Research and Development, U.S. Environmental Protection Agency, Research Triangle Park, NC 27711, USA; Sarwar.Golam@epa.gov
[5] Key Laboratory of Marine Chemistry Theory and Technology, Ministry of Education, College of Chemistry and Chemical Engineering, Ocean University of China, Qingdao 266100, China; gpyang@ouc.edu.cn
[6] Department of Atmospheric Chemistry and Climate, Institute of Physical Chemistry Rocasolano, CSIC, 28006 Madrid, Spain; a.saiz@csic.es
* Correspondence: yan_zhang@fudan.edu.cn; Tel.: +86-136-3637-2289

Table S1. DMS reactions added into CB05 gas phase chemical mechanism.

No.	Reaction	Reference
1	DMS + OH = SO_2+ … (abstraction channel)	Sander et al. (2016)
2	DMS + OH = 0.75*SO_2+ 0.25*MSA+ … (addition channel)	Sander et al. (2016)
3	DMS + NO_3 = SO_2+ …	Sander et al. (2016)
4	DMS + BrO = 0.75*SO_2+ 0.25*MSA+ …	Atkinson et al. (2006)
5	DMS + IO = 0.75*SO_2+ 0.25*MSA+ …	Atkinson et al. (2006)
6	DMS + ClO = 0.75*SO_2+ 0.25*MSA+ …	Atkinson et al. (2006)
7	DMS + Cl = 0.86*SO_2+ …	Atkinson et al. (2006)

Table S2. Summary of historical cruise surveys for DMS concentration in seawater.

	East China Sea	Bohai Sea and Yellow Sea	Changjiang (Yangtze) Estuary
Spring	April-May, 2009<br>May, 2016<br>April-May, 2017	April-May, 2009<br>April, 2010<br>April-May, 2017	March, 2014<br>March, 2015<br>March, 2016<br>May, 2017
Summer	July, 2011<br>June, 2012<br>June, 2013<br>June, 2015<br>June, 2016	June-July, 2011<br>June-July, 2013<br>August, 2015	July, 2014<br>July, 2015<br>June, 2016<br>July, 2016<br>July, 2017
Autumn	October, 2012<br>October-November, 2013	September, 2010<br>September, 2011	

	October-November, 2015<br>September-October, 2016	November, 2011<br>November, 2012	
Winter	December, 2011-January, 2012	December, 2009<br>December, 2011-January, 2012<br>January-February, 2017<br>December, 2017	February, 2017

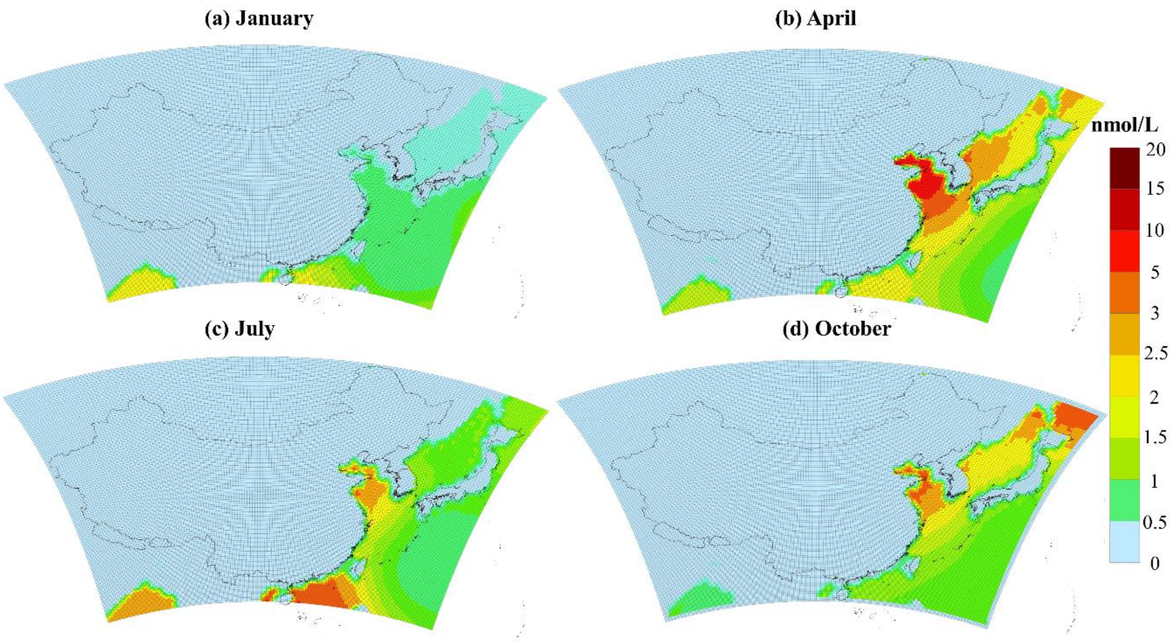

Figure S1. DMS concentration in seawater from the Global Surface Seawater DMS in (a) January, (b) April, (c) July and (d) October .

(a) Pudong Station, SO_2

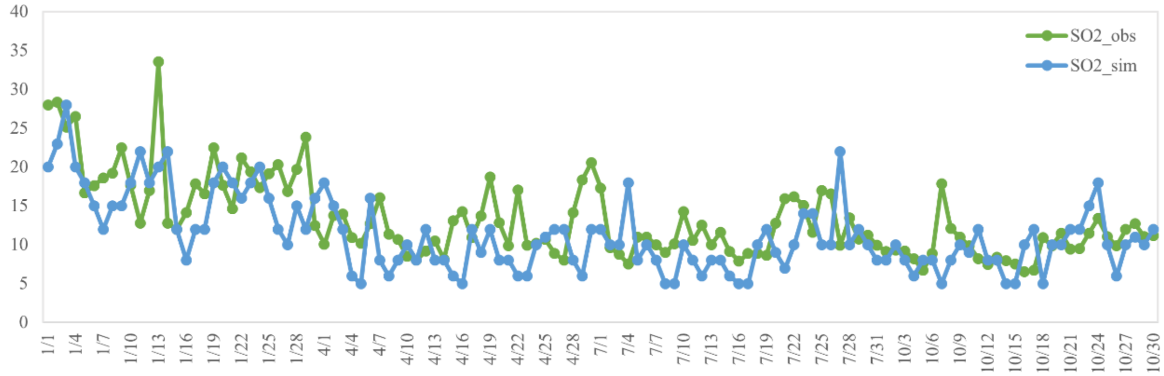

(b) Pudong Station, NO_2

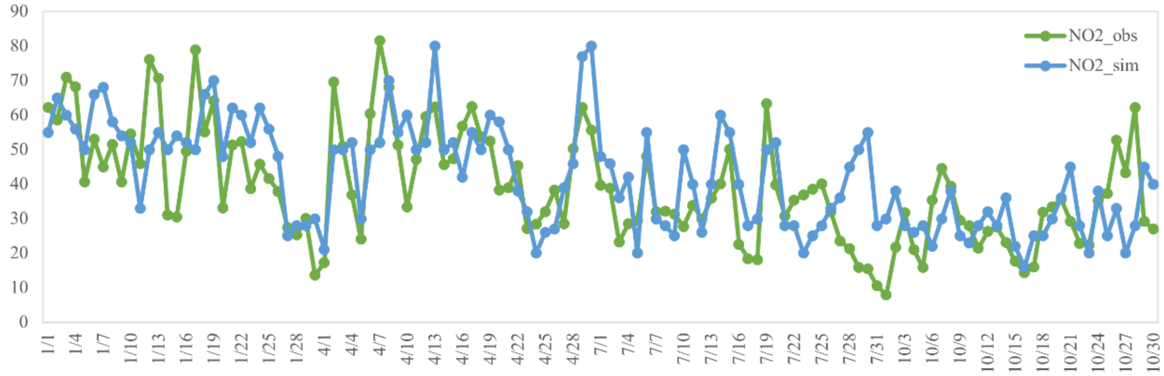

(c) Pudong Station, O_3

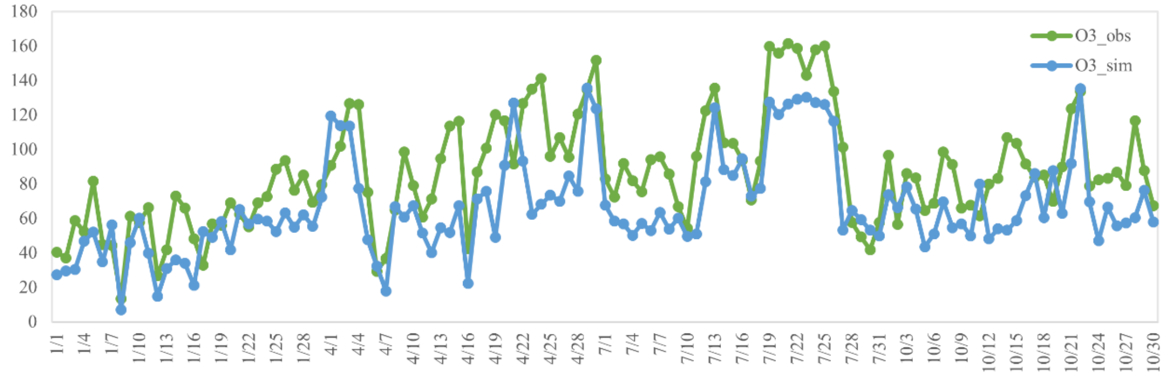

(d) Pudong Station, $PM_{2.5}$

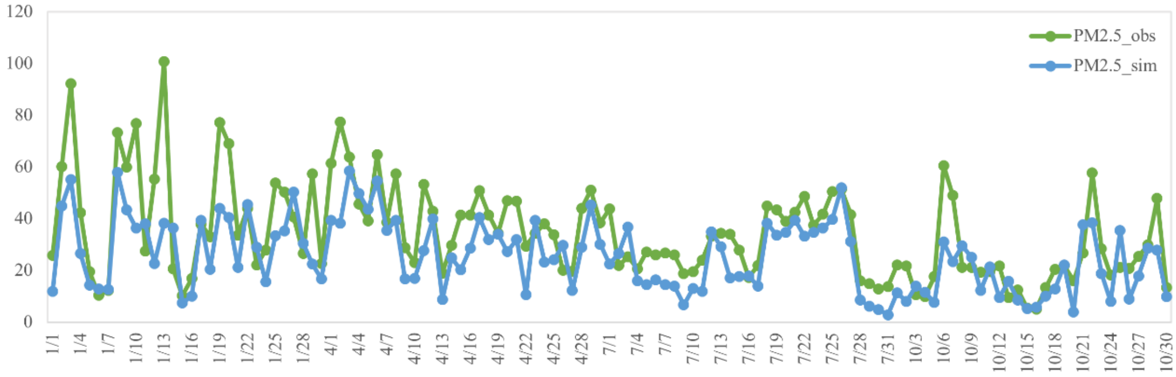

(e) Zhoupu Station, SO_2

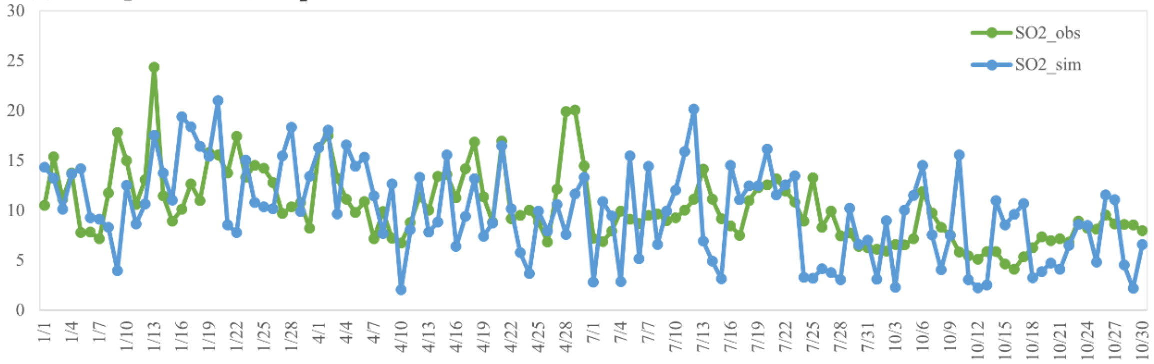

(f) Zhoupu Station, NO_2

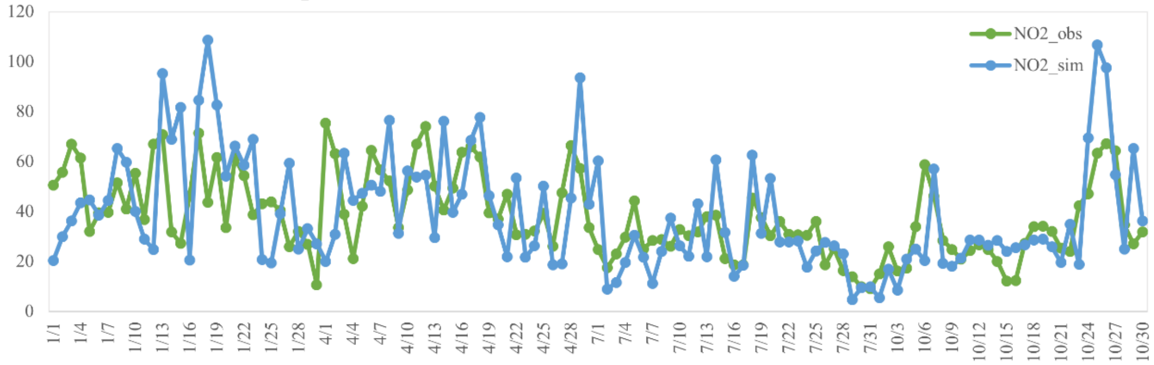

(g) Zhoupu Station, O_3

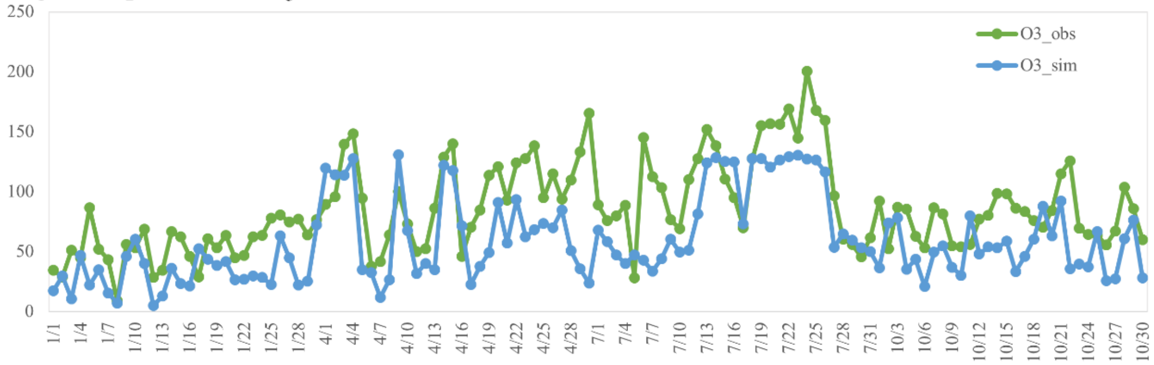

(h) Zhoupu Station, $PM_{2.5}$

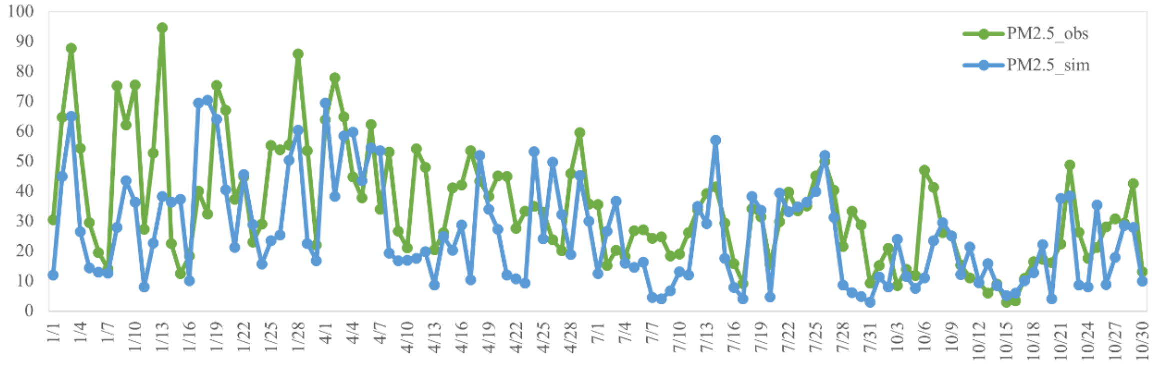

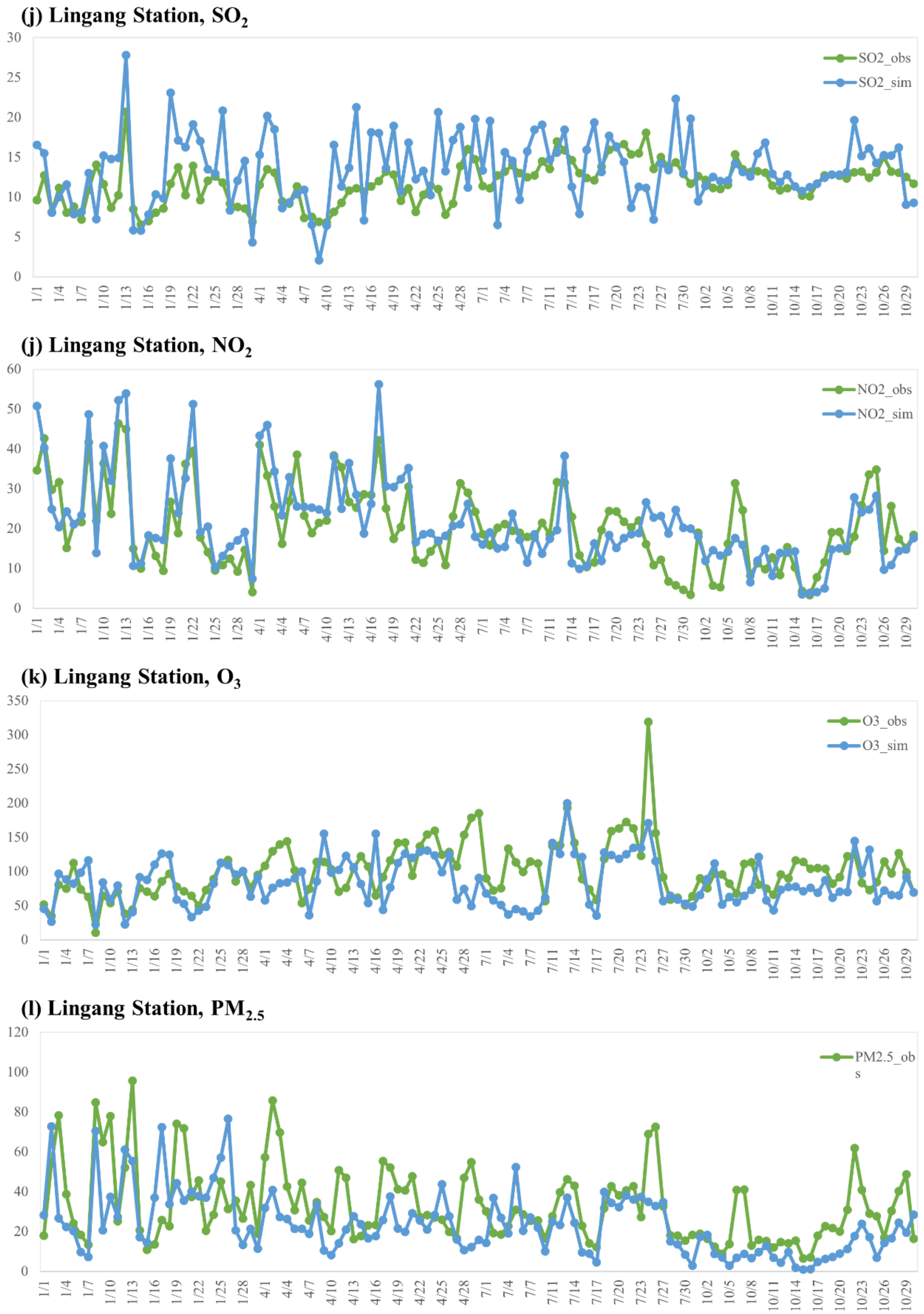

Figure S2. Comparison of observations and simulation results of four major atmospheric pollutants (SO_2, NO_2, O_3 and $PM_{2.5}$) at Pudong Station (a, b, c, d), Zhoupu Station (e, f, g, h), and Lingang Station (i, j, k, l) in Shanghai.

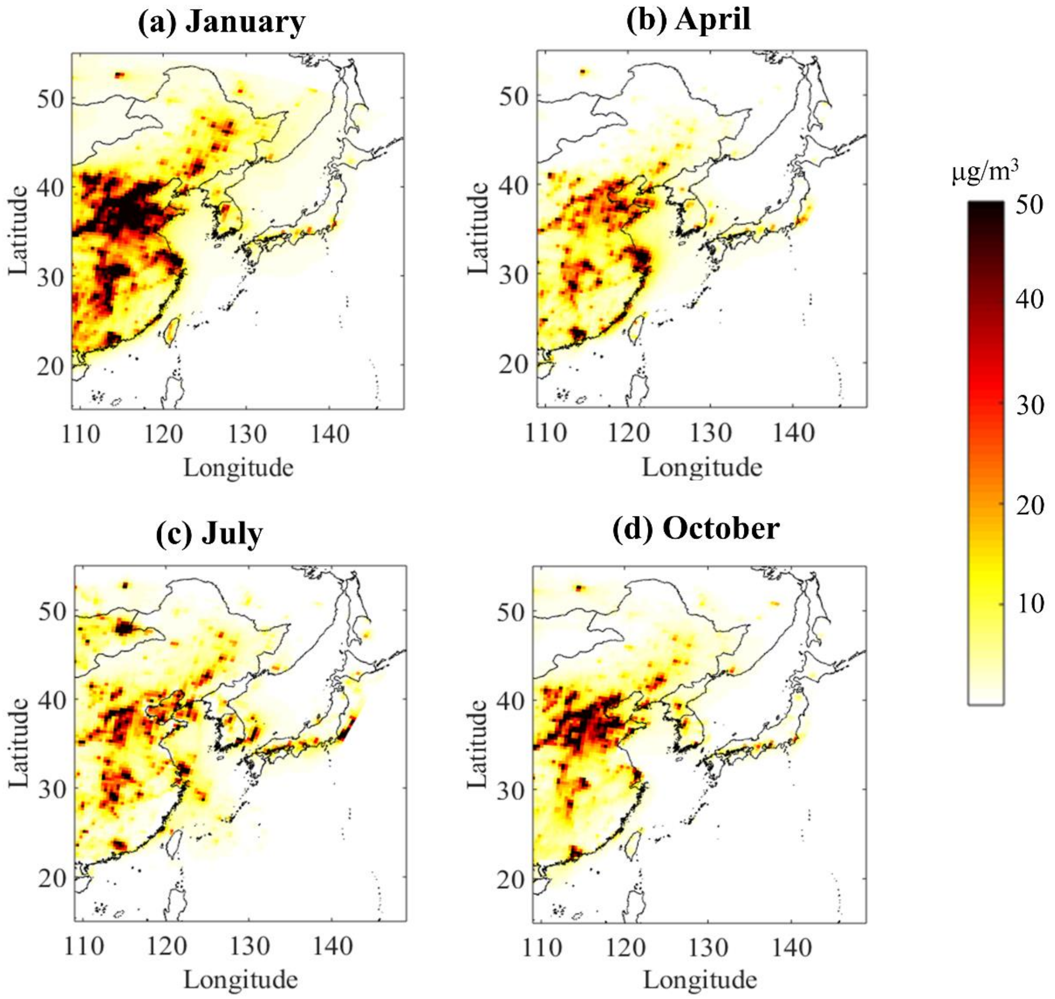

Figure S3. Monthly mean atmospheric SO2 concentration over Chinese seawater from the baseline simulation (without DMS) in four typical months of (a) January, (b) April, (c) July and (d) October.

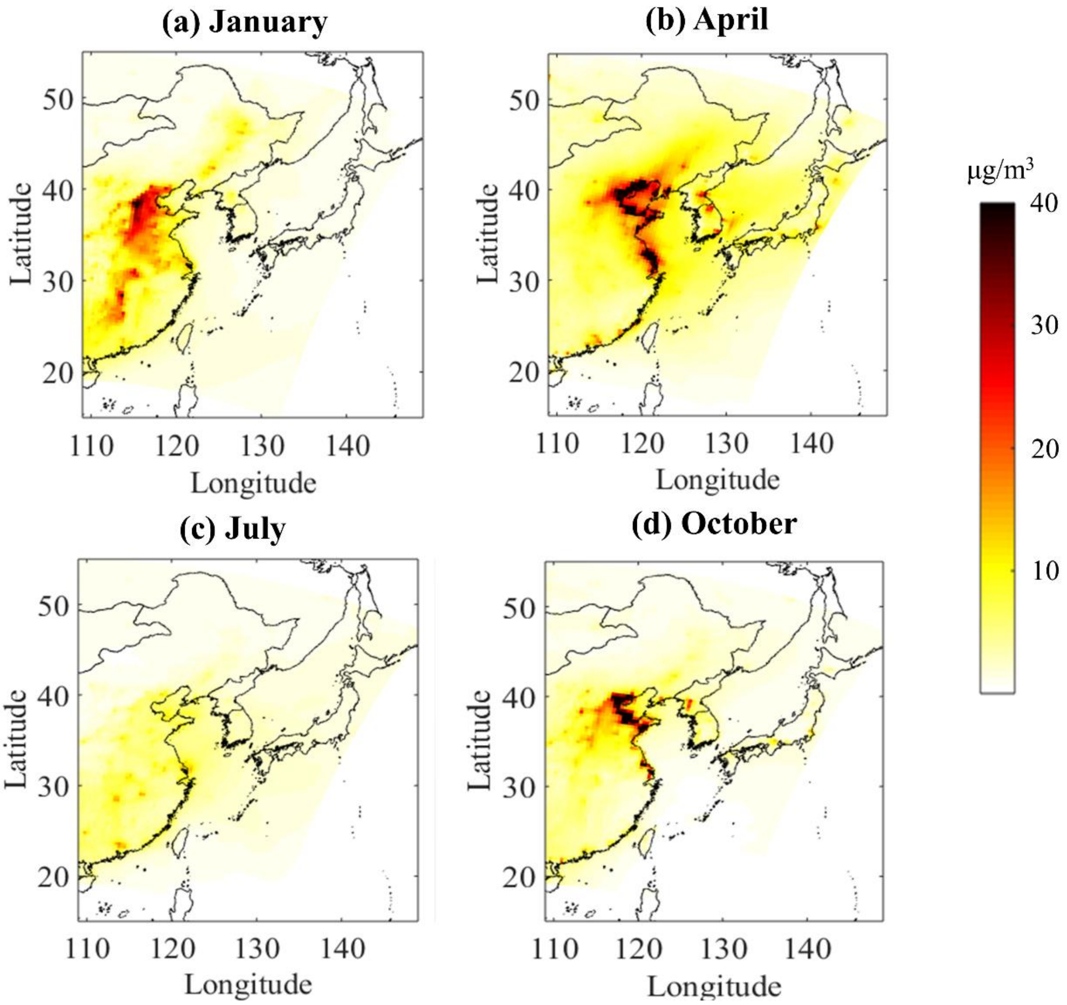

Figure S4. Monthly mean atmospheric SO_4^{2-} concentration over Chinese seawater from the baseline simulation (without DMS) in four typical months of (a) January, (b) April, (c) July and (d) October.
